# Penile hair strangulation: A prodigious cause of pediatric agitation

**DOI:** 10.1002/ccr3.2198

**Published:** 2019-05-17

**Authors:** Jad A. Degheili, Jose M. El‐Asmar, Bilal Aoun

**Affiliations:** ^1^ Division of Urology, Department of Surgery American University of Beirut‐Medical Center Beirut Lebanon; ^2^ Division of Pediatric Nephrology Armand Trousseau University Hospital (APHP) Paris France; ^3^ Division of Pediatric Nephrology, Department of Pediatrics and Adolescent Medicine American University of Beirut‐Medical Center Beirut Lebanon

**Keywords:** hair strangulation, penile amputation, penile necrosis, penis, urological emergency

## Abstract

Penile hair strangulation is secondary to a hair tourniquet effect. Albeit a rare presentation in pediatric emergency units, penile hair strangulation must be cautiously noted in any penile pain and edema, to avoid inevitable damage to external genitalia, including penile necrosis, urethrocutaneous fistula, and even amputation.

A 4‐year‐old boy presented with agitation accompanied by penile swelling and tickling noticed by his father few hours prior to presentation. Physical examination revealed an edematous circumcised penis of adequate length and width concomitant with age. A thin‐lined hair coil was noticed wrapped around the patient's glans penis leading to a bulged, edematous penis (Figure 1).

**Figure 1:**
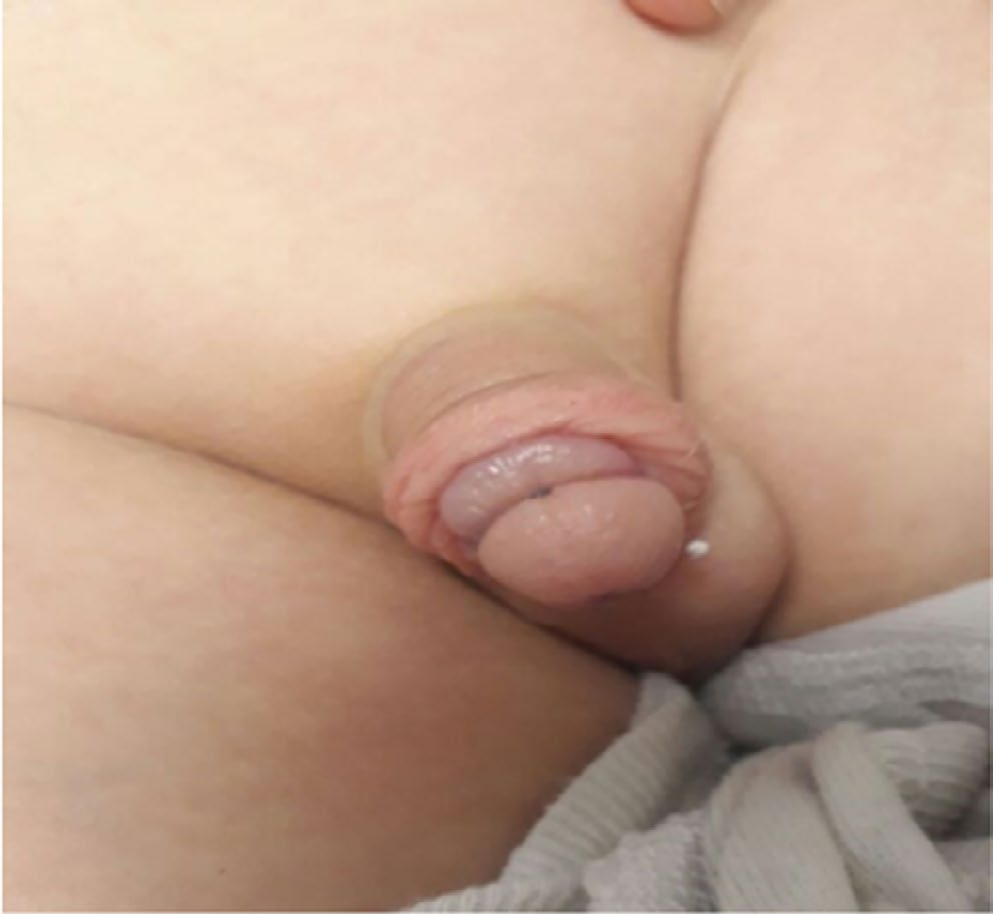
Edematous penis secondary to a hair coil strangulation around the glans penis

Penile tourniquet syndrome, otherwise known as penile hair strangulation, most commonly involve a hair coil as the culprit of compression around the penis.^1^ It is typically hypothesized to be originating from a maternal wet hair that have incidentally found its way to the coronal sulcus. Upon drying, the thin coiled hair loses its elasticity and shortens leading to a set of possible complications that range from simple edema to the most severe scenarios of penile necrosis, ultimately leading to amputation.^2^ It must be considered in the differential for any penile swelling and edema (Table 1).

**Table 1:** Differential diagnosis of neonatal penile swelling

Contact dermatitis
Penile trauma
Paraphimosis
Balanoposthitis (infectious/contact‐induced)
Urethritis
Postinstrumentation, for example, indwelling catheter insertion
Insect bites
Sexual assault
Penile hair strangulation

Management of such cases varies with the severity of injury and ranges from simple detachment of the hair coil to urethroplasty and ventral dartos fascia coverage at the site of defect.^1^

Albeit a rare presentation in pediatric wards, penile hair strangulation must be cautiously noted to avoid any serious damage to external genitalia.

## CONFLICT OF INTEREST

None declared.

## AUTHOR CONTRIBUTIONS

All authors equally accessed the data and contributed to the preparation of the manuscript. JAD, JME‐A, and BA: were equally responsible for making and performing treatment decisions. BA: reviewed the manuscript for critical intellectual content and had the final approval.
